# Editorial: Ageing and Carcinogenesis

**DOI:** 10.3389/fonc.2022.849127

**Published:** 2022-02-16

**Authors:** Raúl A. Ruggiero, Paula Chiarella, Carlos María Galmarini

**Affiliations:** ^1^ Department of Experimental Oncology, IMEX-National Council of Scientific and Technical Research (CONICET), National Academy of Medicine, Buenos Aires, Argentina; ^2^ Topazium Artificial Intelligence, R&D Department, Madrid, Spain

**Keywords:** editorial topic, ageing, carcinogenesis, senescence, cancer

Despite their wide differences (1), most theories about cancer proposed during the past century agree that cancer is a biological nonsense for the organism in which it originates since cancer cells are believed to be - relatively - autonomous, meaning that they are not subject to the rules and regulations that control normal cell proliferation and differentiation in the organism. However, evidence collected throughout the animal kingdom (2), suggest that cancer seems to arise only in organs and tissues that have experienced diminished or lost regenerative ability. In these organs, any injury causing loss of cells or cellular function cannot be compensated by cellular division and in consequence, the original size and function cannot be restored. We suggest that this situation induces a crisis, which, through putative danger signals resulting from retardation of tissue repair, acceleration of cell loss and functional compromise, might promote some degree of variability in the remaining live but arrested cells of the injured organ. The outcome of this situation would be the emergence, by chance, of a genetically or epigenetically modified cell variant bearing mitotic ability to respond to the reparative signal. This new variant would begin to divide and if it were poorly functional or non-functional, the organ would be numerically but not functionally restored. In consequence, it would not score the regeneration as effective and it would continue to send mitotic signals to restore the lost or diminished organ function. As a result, the new variant would grow over and over and the outcome would be a tumor. According to this hypothesis that has called the hypothesis of the biological sense of cancer (3, 4), cancer would not be an autonomous entity disobeying the mechanisms controlling cell proliferation; in fact, in the injured organ which has its regenerative ability lost or diminished, a tumor cell would be the only one able to respond to the demand to proliferate surrounded by “normal” but arrested cells that cannot respond to that signal. According to this interpretation, cancer would have a profound biological sense: it would be the ultimate attempt to restore organ functions and structures that have been lost or altered by aging and noxious environmental agents. However, unlike normal structures, cancer would have no physiological value, because the usually poor or non-functional nature of its cells would make their reparative task unattainable. All conventional therapies against cancer attempt to kill all tumor cells (5–7). However, according to this hypothesis, the problem might not be solved even if all the tumor cells were eradicated. In effect, if the organ failure remained, new tumor cells would emerge and the tumor would reinitiate its progressive growth in response to the permanent regenerative signal of the non-restored organ. An efficient anti-cancer therapy should combine an attack against the tumor cells themselves with the correction of the organ failure, which, according to this hypothesis, would be in the core of the cancer problem.

Therefore, the in-deep understanding of the role of senescence (incapacity of cells to divide even in the face of physiological reparative signals) in carcinogenesis may become an important research field that might impact in the development of new therapies against cancer.

A review by Yang et al. summarizes a double role of senescence in cancer. Firstly, at cellular level, a senescent cell may be a barrier to tumor development because the constitutive difficulty of a senescent cell to initiate cellular proliferation to form a tumor. On the other hand, at tissue level, the accumulation of senescent cells in a tissue or in an organ may induce organismal ageing and simultaneously contribute to the origin and progression of cancer. In fact, several components of the senescence-associated secretory phenotype (SASP) may induce inflammatory conditions that can directly or indirectly promote tumor growth, invasion and metastasis, and tumor vascularization. In addition, recent studies have found that senescent cells are tumor promoters by up-regulating p38MAPK and MAPK/ERK signaling.

The above considerations strongly suggest that modulating SASP or eliminating senescent cells by the use of senolytic drugs may be beneficial for cancer prevention and treatment.

It is known that cellular senescence is triggered by different factors, including, among others, telomere shortening and DNA damage. Zhu et al. showed that the balance of ubiquitination and deubiquitination, that play different roles in regulating protein stability and cellular homeostasis, is another inducer of cellular senescence. Senescent cells are classically identified by their expression of beta-galactosidase. Guan et al. mentioned others tools to explore senescence: genomic molecular biomarkers, including tumor copy number (CN) alteration (CNA) burden, genomic rearrangement signatures, and mutational signatures, where they have been reported as independent molecular prognostic makers in different types of cancer. The authors demonstrated that CN signatures manifest a close correlation to the relevant mutational processes in tumor staging of upper tract urothelial carcinomas (UTUCs), where is relatively difficult to assert accurately before surgery. They used it as a tool to explore their clinical significance of molecular stratification in UTUC, providing a basis for clinical studies that evaluate therapeutic interventions and prognosis.

As we previously introduced, they are different way to study cellular senescence. Bhaskaran et al. investigated how immune system cells (Treg, MDSC) and molecules as Dectin-1, can be dys-regulated in human oral squamous cell carcinoma (OSCC) tissues and a mouse model (4-Nitroquinoline 1-oxide oral carcinogenesis). In summary, the study sheds new light on immuno-oncological events at tumor immune microenvironment, providing some key insights into immune evasion mechanisms. By linking the pro-tumorigenic role of dectin-1 signaling, MDSC and Tregs with an increased predisposition of the aging oral mucosa to tumor development, the study also provides important information on the mechanisms of increased susceptibility of the aging population to oral cancer.

All of these works may stimulate new investigations aimed to understand the complex mechanisms underlying the relationship among organismal ageing, cellular senescence and cancer.

## Author Contributions

All authors listed have made a substantial, direct, and intellectual contribution to the work and approved it for publication.

## Conflict of Interest

The authors declare that the research was conducted in the absence of any commercial or financial relationships that could be construed as a potential conflict of interest.

## Publisher’s Note

All claims expressed in this article are solely those of the authors and do not necessarily represent those of their affiliated organizations, or those of the publisher, the editors and the reviewers. Any product that may be evaluated in this article, or claim that may be made by its manufacturer, is not guaranteed or endorsed by the publisher.
